# Monosegmental anterior column reconstruction using an expandable vertebral body replacement device in combined posterior–anterior stabilization of thoracolumbar burst fractures

**DOI:** 10.1007/s00402-018-2926-9

**Published:** 2018-04-06

**Authors:** Richard A. Lindtner, Max Mueller, Rene Schmid, Anna Spicher, Michael Zegg, Christian Kammerlander, Dietmar Krappinger

**Affiliations:** 1 Department of Trauma Surgery, Medical University of Innsbruck, Anichstraße 35, 6020 Innsbruck, Austria; 2 Department of General, Trauma and Reconstructive Surgery, Ludwig Maximilian University Munich, Marchioninistrasse 15, 81377 Munich, Germany

**Keywords:** Spinal injury, Thoracolumbar fracture, Burst fracture, Combined posterior–anterior stabilization, 360° fusion, Monosegmental, Vertebral body replacement, Anterior column reconstruction

## Abstract

**Introduction:**

In combined posterior–anterior stabilization of thoracolumbar burst fractures, the expandable vertebral body replacement device (VBRD) is typically placed bisegmentally for anterior column reconstruction (ACR). The aim of this study, however, was to assess feasibility, outcome and potential pitfalls of monosegmental ACR using a VBRD. In addition, clinical and radiological outcome of monosegmental ACR was related to that of bisegmental ACR using the same thoracoscopic technique.

**Methods:**

Thirty-seven consecutive neurologically intact patients with burst fractures of the thoracolumbar junction (T11–L2) treated by combined posterior–anterior stabilization were included. Monosegmental ACR was performed in 18 and bisegmental ACR in 19 patients. Fracture type and extent of vertebral body comminution were determined on preoperative CT scans. Monosegmental and bisegmental kyphosis angles were analyzed preoperatively, postoperatively and at final radiological follow-up. Clinical outcome was assessed after a minimum of 2 years (74 ± 45 months; range 24–154; follow-up rate 89.2%) using VAS Spine Score, RMDQ, ODI and WHOQOL-BREF.

**Results:**

Monosegmental ACR resulted in a mean monosegmental and bisegmental surgical correction of − 15.6 ± 7.7° and − 14.7 ± 8.1°, respectively. Postoperative monosegmental and bisegmental loss of correction averaged 2.7 ± 2.7° and 5.2 ± 3.7°, respectively. Two surgical pitfalls of monosegmental ACR were identified: VBRD positioning (1) onto the weak cancellous bone (too far cranially to the inferior endplate of the fractured vertebra) and (2) onto a significantly compromised inferior endplate with at least two (even subtle) fracture lines. Ignoring these pitfalls resulted in VBRD subsidence in five cases. When relating the clinical and radiological outcome of monosegmental ACR to that of bisegmental ACR, no significant differences were found, except for frequency of VBRD subsidence (5 vs. 0, *P* = 0.02) and bisegmental loss of correction (5.2 ± 3.7° vs. 2.6 ± 2.5°, *P* = 0.022). After exclusion of cases with VBRD subsidence, the latter did not reach significance anymore (4.9 ± 4.0° vs. 2.6 ± 2.5°, *P* = 0.084).

**Conclusions:**

This study indicates that monosegmental ACR using a VBRD is feasible in thoracolumbar burst fractures if the inferior endplate is intact (incomplete burst fractures) or features only a single simple split fracture line (burst-split fractures). If the two identified pitfalls are avoided, monosegmental ACR may be a viable alternative to bisegmental ACR in selected thoracolumbar burst fractures to spare a motion segment and to reduce the distance for bony fusion.

## Introduction

**Fig. 1:**
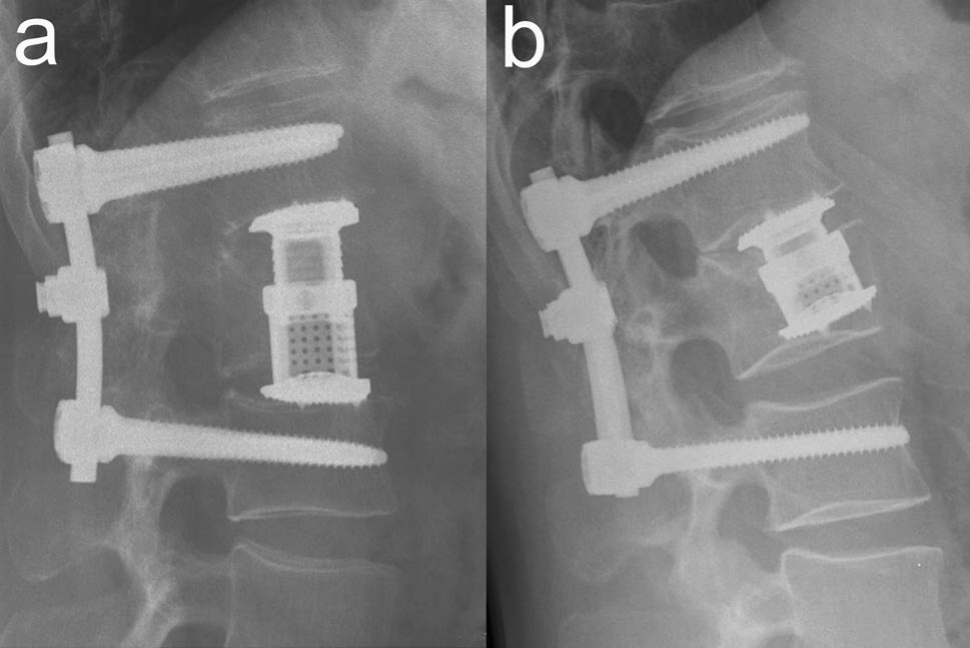
Anterior column reconstruction (ACR) using a vertebral body replacement device (VBRD). In bisegmental ACR (**a**), the VBRD is placed bisegmentally between the superior endplate of the caudad intact vertebra and the inferior endplate of the cephalad intact vertebra. In monosegmental ACR (**b**), the VBDR is placed monosegmentally between the inferior endplate of the fractured vertebra and the inferior endplate of the cephalad intact vertebra

Combined posterior–anterior stabilization using an expandable vertebral body replacement device (VBRD) for anterior column reconstruction (ACR) has been advocated and successfully used by several authors for unstable thoracolumbar burst fractures with significant vertebral body comminution [1–8]. This approach allows for posterior reduction and stabilization (with or without neural decompression) as well as for immediate restoration of the structural strength and load-bearing capacity of the anterior column; it offers high primary biomechanical stability [9–13] and is associated with only minimal postoperative loss of kyphosis correction [1–4]. Typically, the VBRD is placed bisegmentally between the superior endplate of the caudad intact vertebra and the inferior endplate of the cephalad intact vertebra (bisegmental ACR, Fig. 1a). This results in fusion of two motion segments and requires partial resection of the fractured vertebral body (including the superior and inferior endplate) as well as of the adjacent cephalad and caudad intervertebral discs.

In a substantial portion of burst fractures with significant vertebral body comminution, however, the inferior endplate is intact or shows a simple split fracture line only. In this situation, the VBRD may be sufficiently anchored in the intact caudal part of the fractured vertebra and thus may be implanted monosegmentally between the inferior endplate of the fractured vertebra and the inferior endplate of the cephalad intact vertebra (monosegmental ACR, Fig. 1b). As opposed to bisegmental ACR, monosegmental ACR offers the advantages of (1) sparing one spinal motion segment in these predominantly young patients and of (2) reducing the distance for osseous bridging to achieve bony fusion. However, there are no studies to date that have systematically assessed the feasibility and outcome of monosegmental ACR using a VBRD in thoracolumbar burst fractures.

The aim of the present study, therefore, was to assess the feasibility as well as the radiological and clinical outcome of thoracoscopic monosegmental ACR using an expandable VBRD in combined posterior–anterior stabilization of thoracolumbar burst fractures (T11–L2) without neurological deficits. Furthermore, the outcome of monosegmental ACR was related to that of bisegmental ACR using the same thoracoscopic technique. We hypothesized that (1) monosegmental ACR is feasible in burst fractures with a largely intact inferior end plate and that (2) radiological and clinical outcome is comparable to that of bisegmental ACR.

## Materials and methods

### Patients

The study was approved by the institutional review board and written informed consent was obtained from all individual participants included in the study. Inclusion criteria were defined as follows: (1) burst fracture of the vertebral body (subtype A3 or A4 according to the AOSpine Thoracolumbar Spine Injury Classification System [14] and group A3 fractures according to the Magerl classification system [15]) of the thoracolumbar junction (T11–L2) treated by combined posterior–anterior stabilization with mono- or bisegmental ACR using an expandable VBRD (Synex™, Synthes Inc., Bettlach, Switzerland or Hydrolift®, Aesculap AG, Tuttlingen, Germany); (2) age > 18 and < 65 years; and (3) absence of neurological deficits [American Spinal Injury Association (ASIA) Impairment Scale (AIS) grade E]. Exclusion criteria were age < 18 and > 65 years; vertebral body fractures with intact posterior wall (subtype/group A1 and A2 according to the AOSpine Thoracolumbar Spine Injury Classification System and Magerl classification system); level of fracture above T11 or below L2; neurological deficit (AIS grade A–D); corrective surgery of posttraumatic deformity; pathological and osteoporotic fractures; insufficient German language skills to complete the questionnaires.

From January 1, 2000 to December 31, 2011, 84 patients with thoracic or lumbar burst fractures have been treated by combined posterior–anterior stabilization using an expandable VBRD for ACR. Of these 84 patients, 37 patients (20 males and 17 females; mean age 47.4 ± 9.9 years at the time of surgery, range 26–62 years) met all inclusion and exclusion criteria for this study. Eighteen patients underwent monosegmental and 19 underwent bisegmental ACR. The decision for either monosegmental or bisegmental ACR was based on the surgeon’s preference and judgment. Monosegmental ACR was performed by four experienced surgeons. The same four surgeons also performed the bisegmental ACR procedures. Of the 47 patients excluded, 17 sustained a fracture above or below TH11–L2, 19 presented with neurological deficits, four underwent corrective surgery of posttraumatic deformity and seven had insufficient German language skills to complete the questionnaires.

### Surgical technique

All study patients underwent combined posterior–anterior stabilization with thoracoscopic anterior column reconstruction using an expandable VBRD as described by Knop et al. [16]. Overall the procedure was carried out as a one-staged procedure in 26 patients and as a two-stage procedure in 11 patients (Table 2). In a first step, posterior reduction and bisegmental posterior instrumentation using an angular stable pedicle screw system including a crosslink (USS™ pedicle screw System, Synthes Medical, Oberdorf, Switzerland) was performed with the patient in the prone position. In a second step, the anterior column was reconstructed via four standardized portals with the patient in a right lateral decubitus position and with one-lung ventilation. For monosegmental anterior column reconstruction, the VBRD was monosegmentally implanted and expanded between the inferior endplate of the fractured vertebra and the inferior endplate of the cephalad intact vertebra (Fig. 1b). For bisegmental anterior column reconstruction, the VBRD was bisegmentally implanted and expanded between the superior endplate of the caudad intact vertebra and the inferior endplate of the cephalad intact vertebra (Fig. 1a).

Postoperative management was the same in both groups: after removal of the chest tube on the first or second postoperative day, the patients were mobilized without further bracing under guidance of a physiotherapist as pain and general status allowed. Return to sport, physical work and heavy lifting was permitted after 3 months.

### Radiographic measurements

At admission, computed tomography scans and spinal radiographs with the patient supine were obtained from all patients. Upright radiographs were performed after postoperative mobilization and at the follow-up visits. For radiographic evaluation, the monosegmental kyphosis angle (MKA) and bisegmental kyphosis angle (BKA) were determined on pre- and postoperative lateral radiographs as well as on lateral radiographs at time of final follow-up. The MKA was defined as the angle between the superior endplate of the cephalad intact vertebra and the inferior endplate of the fractured vertebra measured by the Cobb method. The BKA was defined as the angle between the superior endplate of the cephalad intact vertebra and the inferior endplate of the caudad intact vertebra measured by the Cobb method. In patients with bisegmental anterior column reconstruction only the BKA was determined as MKA measurement is not feasible. Kyphotic angles were assigned positive values, while lordotic angles were assigned negative values. Mono- and bisegmental surgical correction was calculated by subtracting the preoperative MKA and BKA from the postoperative MKA and BKA, respectively. Mono- and bisegmental loss of correction was calculated by subtracting the postoperative MKA and BKA from the MKA and BKA at final radiological follow-up, respectively. In patients who underwent implant removal of the dorsal instrumentation, MKA and BKA were additionally determined on upright radiographs taken prior to implant removal, and were compared to the respective angles measured on postoperative radiographs after initial surgery as well as on radiographs at final radiological follow-up. VBRD subsidence was determined by reviewing the VBRD position on all available postoperative radiographs.

Preoperative multiplanar CT reconstructions were reviewed to determine fracture type according to the AOSpine Thoracolumbar Spine Injury Classification System as well as the Magerl classification system. Moreover, the extent of vertebral body comminution was assessed on sagittal CT reconstructions. To this purpose, the sagittal cross-sectional area of the fractured vertebral body was subdivided into an upper, middle and caudal third by two horizontal lines to analyze if comminution involves the upper third only, the upper and middle third or all three thirds. Two independent observers who were not involved with the treatment of these patients evaluated all radiographs and computed tomography scans.

### Clinical outcome assessment

The clinical outcome was assessed after a minimum follow-up of 2 years with use of a postal questionnaire including the following validated clinical outcome measurement instruments:

Visual Analogue Scale (VAS) Spine Score [17], Roland–Morris Disability Questionnaire (RMDQ) and Oswestry Disability Index (ODI) were used to assess back-specific pain and function at time of final follow-up. Patients were furthermore asked to complete the VAS Spine Score to the best of their knowledge for the time prior to the injury. Quality of life was evaluated using the WHOQOL-BREF, an abbreviated 26-item version of the World Health Organization Quality of Life assessment instrument (WHOQOL-100). WHOQOL-BREF is scored in four domains: physical capacity (seven items), psychological well-being (six items), social relationship (three items), and environment (eight items).

### Statistical analysis

IBM SPSS version 24 (IBM Corp, Armonk, NY, United States) was used for statistical analysis. Metric scaled data are reported as arithmetic mean ± standard deviation and categorical data as absolute frequency and percentage distribution. Depending on the distribution form, a *t* test for independent variables or a nonparametric Mann–Whitney U test was used. The distribution form was determined using the Kolmogorov–Smirnov test. A Pearson Chi-Square test or a Fisher’s exact test was used for analysis of categorical data. The level of significance was set at *P* < 0.05.

## Results

### Patient characteristics

During the 12-year period, a total of 37 neurologically intact patients with thoracolumbar burst fractures (T11-L2) managed by combined posterior–anterior stabilization using an expandable vertebral body replacement device (VBRD) for anterior column reconstruction (ACR) met all inclusion and exclusion criteria. Of these 37 patients, 18 patients (49%) underwent monosegmental ACR and 19 patients (51%) underwent bisegmental ACR.

Patient characteristics and surgery-related data are summarized in Tables 1 and 2, respectively. The two groups did not significantly differ in age, sex, mechanism of injury, level of fracture, injury type or any variable of surgery-related data except for time to implant removal (Table 2). However, the degree of vertebral body injury classified according to the AOSpine Thoracolumbar Spine Injury Classification System [A3 (incomplete burst) vs. A4 (complete burst)] and according to the Magerl classification system [A3.1 (incomplete burst) vs. A3.2 (burst-split) vs. A3.3 (complete burst)] as well as the extent of vertebral body comminution were significantly lower in patients with monosegmental ACR than in those with bisegmental ACR (Table 1). The majority of injuries resulted from sports accidents [16/37 (43%)] and falls from height [14/37 (38%)], and most commonly involved the L1 [22/37 (60%)] and T12 [12/37 (32%)] vertebra (Table 1). In 56% (10/18) of monosegmental and 84% (16/19) of bisegmental ACR, posterior stabilization and ACR were performed as one-staged procedure. The mean length of hospital stay was 19.5 ± 16.3 days. No patient deteriorated neurologically during hospital stay or follow-up period.

**Table 1:** Patient characteristics

	Monosegmental ACR	Bisegmental ACR	*P* value
*n*	18	19	
Age (years)	44.6 ± 7.8	49.9 ± 11.1	0.10
Male sex	10	10	0.99
Mechanism of injury			
Sports accident	10	6	0.15
Fall from height	7	7	
Traffic accident	1	2	
Other	0	4	
Level of fracture			
T11	0	1	0.21
T12	4	8	
L1	12	10	
L2	2	0	
AOSpine type			
Type A	14	11	0.26
Type B	4	6	
Type C	0	2	
AOSpine VB			
A3	6 (33%)	1 (5%)	**0.042**
A4	12 (67%)	18 (95%)	
Magerl VB			
A3.1	6 (33%)	1 (5%)	**0.002**
A3.2	9 (50%)	4 (21%)	
A3.3	3 (17%)	14 (74%)	
Extent of VB comminution			
Upper third involved	1 (5.6%)	1 (5.3%)	**0.002**
Upper two-third involved	14 (77.8%)	4 (31.6%)	
All three thirds involved	3 (16.7%)	14 (63.2%)	

Bold values indicate *P* < 0.05

*ACR* anterior column reconstruction, *AOSpine type* injury type according to the AOSpine Thoracolumbar Spine Injury Classification System, *AOSpine VB* vertebral body (VB) fracture classified according to the AOSpine Thoracolumbar Spine Injury Classification System, *Magerl VB* vertebral body (VB) fracture classified according to the Magerl classification system

**Table 2:** Surgery-related data

	Monosegmental ACR	Bisegmental ACR	*P* value
Time from admission to surgery (days)	4.7 ± 7.6	6.7 ± 10.3	0.94
One-staged procedure, *n* (%)	10 (56%)	16 (84%)	0.08
Time from 1st to 2nd surgery (days) of two-staged procedures	8.4 ± 3.2	5.3 ± 3.1	0.19
Length of hospital stay (days)	17.3 ± 10.6	21.5 ± 20.5	0.73
Posterior instrumentation removed, *n* (%)	9 (50%)	6 (32%)	0.33
Time to implant removal (months)	16.7 ± 5.4	24.0 ± 6.8	**0.037**

Bold value indicate *P* < 0.05

*ACR* anterior column reconstruction

### Radiological outcome

The radiological data are summarized in Table 3. Combined posterior–anterior stabilization with monosegmental ACR improved the mean monosegmental kyphosis angle (MKA) from 13.7 ± 9.8° preoperatively to − 1.9 ± 4.7° postoperatively, resulting in a mean monosegmental surgical correction of − 15.6 ± 7.7°. The mean bisegmental kyphosis angle (BKA) was improved from 6.7 ± 11.6° preoperatively to − 7.9 ± 6.6° postoperatively, resulting in a mean bisegmental surgical correction of − 14.7 ± 8.1°. At final radiological follow-up after a mean of 29 months, MKA and BKA were 0.8 ± 5.4° and − 2.8 ± 7.1°, respectively, corresponding to a monosegmental and bisegmental loss of correction of 2.7 ± 2.7° and 5.2 ± 3.7°, respectively.

**Table 3:** Radiological follow-up

	Monosegmental ACR	Bisegmental ACR	*P* value
*n*	18	19	
Preop BKA (°)	6.7 ± 11.6	8.6 ± 9.4	0.60
Postop BKA (°)	− 7.9 ± 6.6	− 6.5 ± 5.1	0.45
Bisegmental surgical correction (°)	− 14.7 ± 8.1	− 15.0 ± 8.9	0.90
BKA at final follow-up (°)	− 2.8 ± 7.1	− 3.6 ± 6.6	0.73
Preop MKA (°)	13.7 ± 9.8	11.8 ± 10.0	0.57
Postop MKA (°)	− 1.9 ± 4.7	na	na
Monosegmental surgical correction (°)	− 15.6 ± 7.7	na	na
MKA at final follow-up (°)	0.8 ± 5.4	na	na
Time to final radiological follow-up (months)	28.5 ± 28.0	39.5 ± 31.6	0.20
Bisegmental loss of correction (°)	5.2 ± 3.7	2.6 ± 2.5	**0.022**
Monosegmental loss of correction (°)	2.7 ± 2.7	na	na
VBRD subsidence	5	0	**0.02**

Bold values indicate *P* < 0.05

*ACR* anterior column reconstruction, *BKA* bisegmental kyphosis angle, *MKA* monosegmental kyphosis angle, *na* not applicable, *VBRD* vertebral body replacement device

**Fig. 2:**
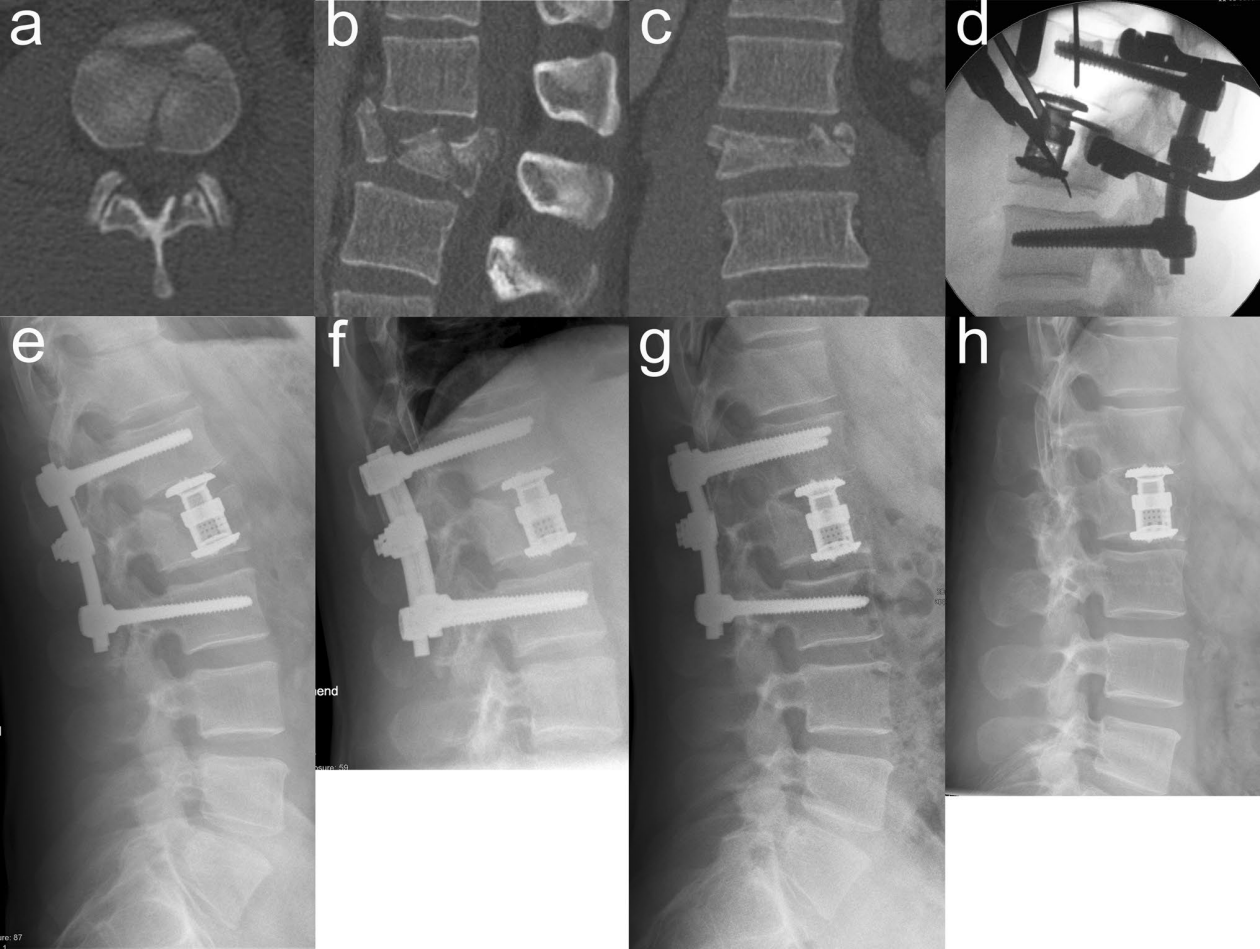
First illustrative case of VBDR subsidence through the inferior endplate after monosegmental ACR. Axial (**a**), sagittal (**b**) and coronal (**c**) CT reconstructions showing a complete burst fracture of L1. The axial CT reconstruction at the level of the inferior endplate of the fractured vertebra (**a**) reveals multiple fracture lines at the inferior end plate. Intraoperative lateral radiograph (**d**) showing monosegmental VBRD placement. Postoperative lateral radiographs at 3 days (**e**), 1 month (**f**), 4 months (**g**) and 34 months (**h** after implant removal) demonstrating VBDR subsidence through the severely compromised inferior endplate and into the adjacent intervertebral disc

**Fig. 3:**
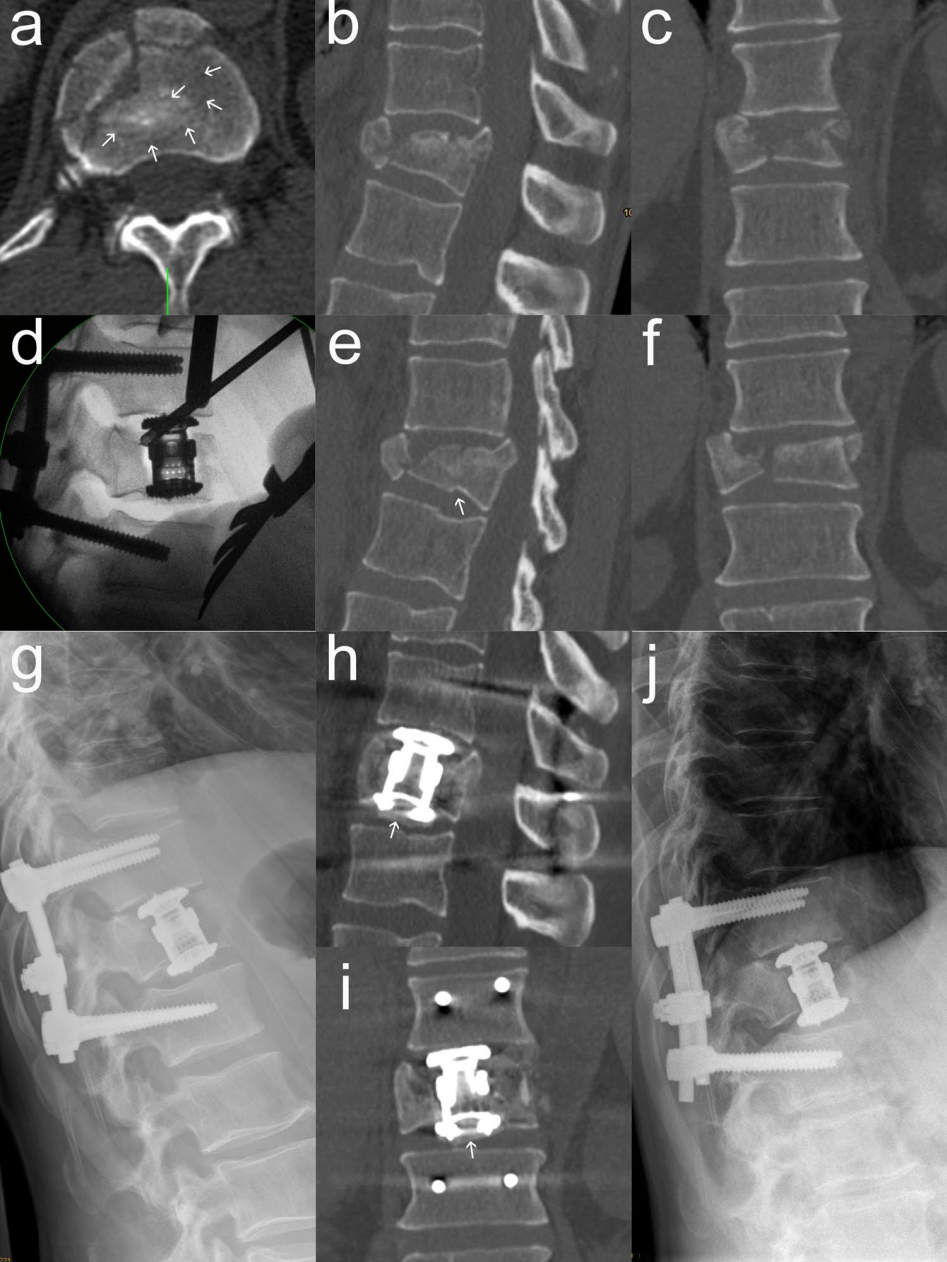
Second illustrative case of VBDR subsidence through the inferior endplate after monosegmental ACR. Multiplanar CT reconstructions in the axial (**a**), median sagittal (**b**), paramedian sagittal (**e**) and coronal (**c, f**) plane showing a complete burst fracture of T12. The fracture may be misinterpreted as a burst-split fracture when analyzing the standard median sagittal and coronal reconstructions only. However, the axial CT reconstruction at the level of the inferior endplate (**a**) as well as the paramedian sagittal reconstruction (**e**) clearly depict multiple additional subtle fracture lines at the inferior endplate (indicated by white arrows). Intraoperative lateral radiograph (**d**) already showing minimal VBRD subsidence after positioning onto the “free floating” central inferior endplate fragment created by the presence of multiple fracture lines. Postoperative lateral radiographs and CT images at 1 week (**g**–**i**) and 14 months (**j**) demonstrating VBDR subsidence through the inferior endplate and into the adjacent intervertebral disc. The central inferior endplate fragment below the VBDR is indicated by white arrows (**h, i**)

**Fig. 4:**
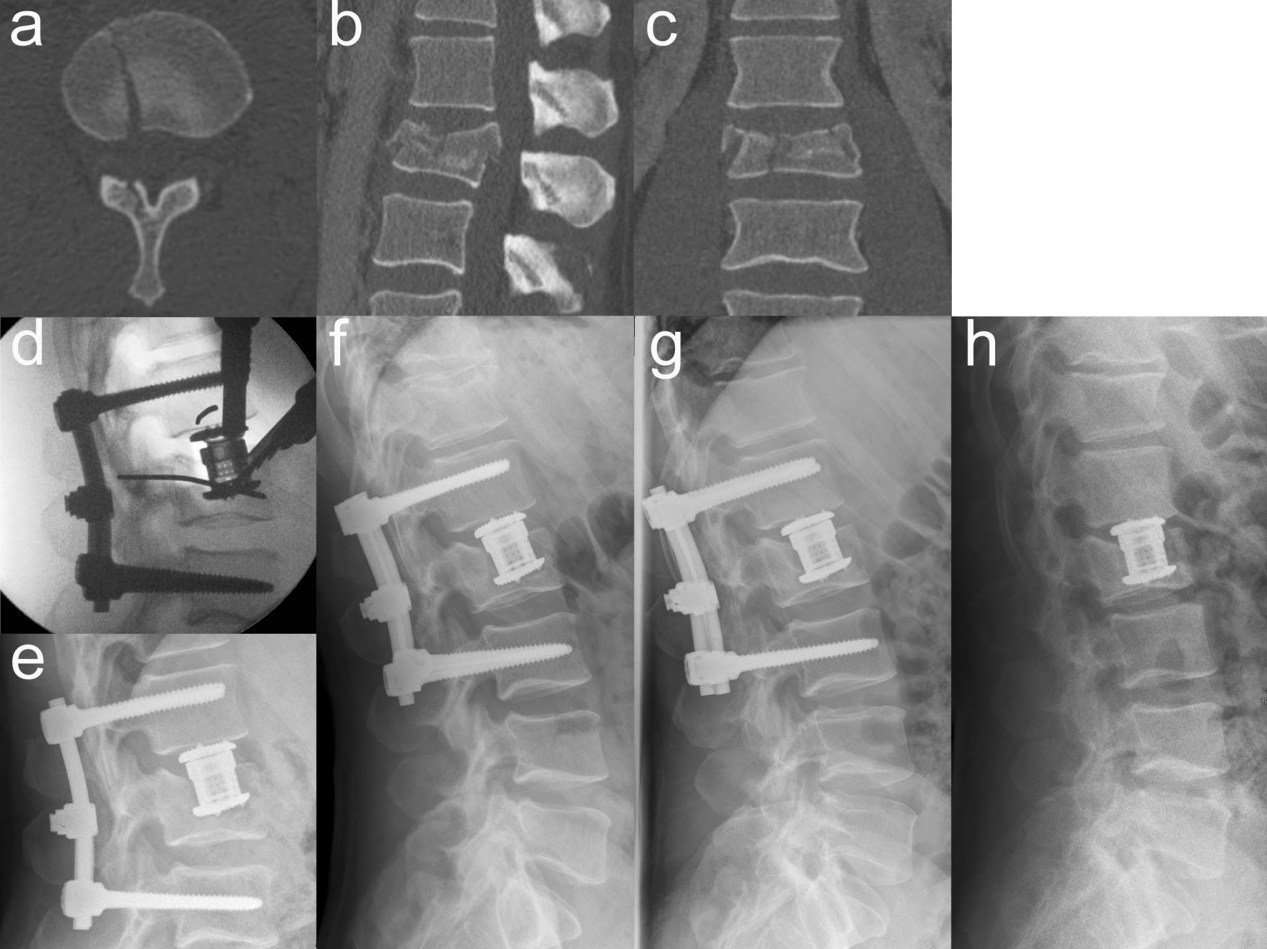
Illustrative case of VBRD subsidence into the cancellous bone after monosegmental ACR. Axial (**a**), sagittal (**b**) and coronal (**c**) CT reconstructions showing a burst-split fracture of L2 and one single split fracture line at the inferior endplate of the fractured vertebra (**a**). Intraoperative lateral radiograph (**d**) demonstrating that the VBRD was placed too far cranially to the inferior endplate of the fractured vertebra and anchored into the weak cancellous bone. Postoperative lateral radiographs at 1 month (**e**), 3 months (**f**), 6 months (**g**) and 13 months (**h** after implant removal): VBDR subsidence occurred between 1 and 3 months after surgery and is clearly evident at 3-month follow-up (**f**)

VBRD subsidence was observed in five patients (Table 4). In three of these patients, the VBRD subsided through the inferior endplate of the fractured vertebra and into the adjacent intervertebral disc (Figs. 2, 3). In the other two patients, the VBRD subsided into the cancellous bone until the inferior endplate was reached (Fig. 4). VBDR subsidence through the inferior endplate was already apparent intraoperatively (one case) or on first radiograph after postoperative mobilization (two cases), whereas VBDR subsidence into the cancellous bone occurred later but became evident on follow-up radiographs within 3 months post surgery. When comparing patients with and without VBRD subsidence after monosegmental ACR, however, differences in monosegmental (4.0 ± 2.6° vs. 2.2 ± 2.7°, *P* = 0.22) and bisegmental (5.8 ± 3.0° vs. 4.9 ± 4.0°, *P* = 0.67) loss of correction did not reach significance.

**Table 4:** Characteristics of patients with VBDR subsidence

	Patient no. 1	Patient no. 2	Patient no. 3	Patient no. 4	Patient no. 5	MWS (*n* = 13)
Type of subsidence	TIE	TIE	TIE	ICB	ICB	
Age (years)	48	38	37	42	50	45.2 ± 8.5
Sex	M	M	M	M	M	
Level	T12	L1	T12	L2	L1	
AOSpine VB	A4	A4	A4	A4	A4	
Magerl VB	A3.3	A3.3	A3.3	A3.2	A3.2	
Revision surgery	No	No	No	No	No	
Posterior instrumentation removed	No	No	No	Yes	Yes	
Preop BKA (°)	10.5	10.9	21.5	2.2	− 0.8	5.9 ± 12.7
Bisegmental surgical correction (°)	− 6.0	− 20.7	− 22.1	− 9.5	− 5.8	− 15.4 ± 8.4
Bisegmental loss of correction (°)	0.7	5.8	6.4	8.2	7.8	4.9 ± 4.0
Preop MKA (°)	19.7	19.5	19.7	10.5	9.9	12.8 ± 11.1
Monosegmental surgical correction (°)	− 15.0	− 26.9	− 14.8	− 12.3	− 9.0	− 15.6 ± 8.3
Monosegmental loss of correction (°)	0.7	6.1	3.1	7.2	2.8	2.2 ± 2.7
Time to final clinical follow-up (months)	27	24	33.9	25	30	77.1 ± 40.4
VAS Spine Score before trauma	84	88	99	100	68	87.0 ± 15.5
VAS Spine Score at final follow-up	61	72	43	86	37	72.1 ± 27.7
Loss in VAS spine score	23	16	56	14	31	14.9 ± 21.6
Roland and Morris disability questionnaire	0	4	15	1	12	3.6 ± 4.9
Oswestry disability index	20	12	30	8	34	18.3 ± 20.8
WHOQOL-BREF physical health	94	69	63	81	44	78.1 ± 20.3
WHOQOL-BREF psychological health	100	81	75	81	63	78.8 ± 15.8
WHOQOL-BREF social relationships	81	100	75	94	75	81.3 ± 20.2
WHOQOL-BREF environment	94	88	69	94	69	82.9 ± 14.9

*MWS* mean value of monosegmental ACR without VBDR subsidence, *TIE* subsidence through inferior endplate, *ICB* subsidence into cancellous bone, *AOSpine VB* vertebral body (VB) fracture classified according to the AOSpine Thoracolumbar Spine Injury Classification System, *Magerl VB* vertebral body (VB) fracture classified according to the Magerl classification system, *WHOQOL-BREF* World Health Organization Quality of Life Assessment Instrument—short form

The posterior instrumentation was removed in half (9/18) of the patients after monosegmental ACR, whereas the other half refused implant removal although advised. When analyzing the patients who underwent implant removal (Table 5), overall monosegmental loss of correction was 3.3 ± 1.9°, with 2.0 ± 2.0° loss occurring until time of implant removal and 1.3 ± 3.0° loss occurring between time of implant removal and final radiological follow-up. Overall bisegmental loss of correction was 6.7 ± 2.9°, with 3.0 ± 3.1° loss occurring until time of implant removal and 3.6 ± 3.2° loss occurring between time of implant removal and final radiological follow-up.

**Table 5:** Radiological follow-up of patients who underwent removal of posterior instrumentation

	Monosegmental ACR	Bisegmental ACR	*P* value
*n*	9 (50%)	6 (32%)	0.33
Preop BKA (°)	6.9 ± 10.7	13.8 ± 11.5	0.26
Postop BKA (°)	− 8.4 ± 5.9	− 5.7 ± 4.7	0.37
Bisegmental surgical correction (°)	− 15.3 ± 8.1	− 20.0 ± 11.6	0.42
BKA at time of implant removal (°)	− 5.4 ± 5.0	− 2.7 ± 4.9	0.34
BKA at final follow-up (°)	− 1.8 ± 5.2	− 1.6 ± 5.3	0.95
Preop MKA (°)	13.3 ± 8.1	na	na
Postop MKA (°)	− 2.4 ± 3.3	na	na
Monosegmental surgical correction (°)	− 15.7 ± 6.9	na	na
MKA at time of implant removal (°)	− 0.4 ± 3.4	na	na
MKA at final follow-up (°)	1.0 ± 3.7	na	na
Time to final radiological follow-up (months)	35.4 ± 35.5	35.3 ± 14.7	0.99
Bisegmental loss of correction (°)	6.7 ± 2.9	4.2 ± 0.8	0.06
Monosegmental loss of correction (°)	3.3 ± 1.9	na	na
Time to implant removal (months)	16.7 ± 5.4	24.0 ± 6.8	**0.037**
Bisegmental loss of correction until implant removal (°)	3.0 ± 3.1	3.0 ± 1.3	0.96
Monosegmental loss of correction until implant removal (°)	2.0 ± 2.0	na	na
Time implant removal to final follow-up (months)	18.7 ± 31.6	11.3 ± 13.5	0.86
Bisegmental loss of correction between implant removal and final follow-up (°)	3.6 ± 3.2	1.2 ± 1.7	0.11
Monosegmental loss of correction between implant removal and final follow-up (°)	1.3 ± 3.0	na	na

Bold value indicate *P* < 0.05

*ACR* anterior column reconstruction, *BKA* bisegmental kyphosis angle, *MKA* monosegmental kyphosis angle, *na* not applicable

Radiological outcome of monosegmental ACR was furthermore related to that of bisegmental ACR (Table 3). Statistical analysis did not reveal any significant differences in the radiological outcome measures between monosegmental and bisegmental ACR, except for frequency of VBRD subsidence (5 vs. 0, *P* = 0.02) and bisegmental loss of correction (5.2 ± 3.7° vs. 2.6 ± 2.5°, *P* = 0.022). After exclusion of cases with VBRD subsidence, the difference in bisegmental loss of correction between monosegmental and bisegmental ACR did not reach significance anymore (4.9 ± 4.0° vs. 2.6 ± 2.5°, *P* = 0.084).

### Patient-reported outcome

Seventeen of 18 patients (94%) with monosegmental ACR were available for clinical follow-up after a mean of 62.7 ± 40.7 months (range 23.8–136.2 months), while one was lost to follow-up (Table 6). Patient-reported pain, function and quality of life were assessed using the VAS Spine Score, Roland and Morris Disability Questionnaire, Oswestry Disability Index and the abbreviated WHO Quality of Life questionnaire (WHOQOL-BREF) (Table 6). The mean loss in VAS Spine Score averaged 18.8 ± 20.8 points.

**Table 6:** Clinical outcome at final follow-up

	Monosegmental ACR	Bisegmental ACR	*P* value
*n*	17	16	
Time to final clinical follow-up (months)	62.7 ± 40.7	86.8 ± 46.2	0.28
VAS spine score before trauma (0–100; 100 = no complaints/pain)	87.2 ± 14.4	91.3 ± 15.4	0.51
VAS spine score at final follow-up (0–100; 100 = no complaints/pain)	68.5 ± 25.7	82.3 ± 17.1	0.13
Loss in VAS spine score	18.8 ± 20.8	9.2 ± 13.5	0.09
Roland and Morris disability questionnaire (0–24; 0 = no complaints/pain)	4.4 ± 5.4	3.0 ± 3.4	0.42
Oswestry disability index (0–100; 0 = no complaints/pain)	19.0 ± 18.2	10.1 ± 9.5	0.17
WHOQOL-BREF physical health (0–100; 100 = best value)	75.8 ± 19.6	82.7 ± 13.3	0.26
WHOQOL-BREF psychological health (0–100; 100 = best value)	79.2 ± 14.8	77.7 ± 13.0	0.76
WHOQOL-BREF social relationships (0–100; 100 = best value)	82.4 ± 17.8	76.3 ± 23.0	0.41
WHOQOL-BREF Environment (0–100; 100 = best value)	82.9 ± 13.9	83.8 ± 13.2	0.74

*ACR* anterior column reconstruction, *WHOQOL-BREF* World Health Organization Quality of Life Assessment Instrument—short form

Patient-reported outcome after monosegmental ACR was furthermore related to that of bisegmental ACR. For bisegmental ACR, the follow-up rate was 16/19 (84%; one patient was lost to follow-up, one died of unrelated causes in the meantime, and one had developed a psychiatric condition and was not able to answer the questionnaires) after a mean of 86.8 ± 46.2 months (range 23.7–154.1 months). Statistical analysis did not reveal any significant differences in the clinical outcome measures between patients with monosegmental and bisegmental ACR (Table 6).

## Discussion

The purpose of this study was to assess the feasibility and outcome of thoracoscopic monosegmental ACR using an expandable VBRD in combined posterior–anterior stabilization of thoracolumbar burst fractures (T11–L2) in neurologically intact patients. Our study indicates that monosegmental ACR is feasible and represents a viable option for anterior spinal reconstruction in thoracolumbar burst fractures provided that the inferior endplate of the fractured vertebra is intact or features only a simple split fracture line. We furthermore found that clinical and radiological outcome of monosegmental ACR was comparable to that of bisegmental ACR, except for frequency of VBRD subsidence (5 vs. 0, *P* = 0.02) and bisegmental loss of correction ACR [5.2 ± 3.7° vs. 2.6 ± 2.5°, (*P* = 0.022)].

To the best of our knowledge, this is the first report systematically evaluating the feasibility and outcome of monosegmental ACR using a VBRD in combined posterior–anterior stabilization of thoracolumbar burst fractures. The optimal treatment of thoracolumbar injuries involving a burst fracture of the vertebral body is still controversial and under debate [18–21]. If combined posterior–anterior stabilization using a VBRD for ACR is considered, monosegmental ACR may be a viable alternative to conventional bisegmental ACR in selected cases. In a substantial portion of burst fractures with significant vertebral body comminution, the caudal endplate is intact (incomplete burst fractures) or features only a single simple split fracture line (burst-split fractures) without significant splaying. In these cases, the VBRD may be sufficiently anchored in the intact caudal part of the fractured vertebra (i.e., monosegmental ACR) to spare one spinal motion segment in this generally young patient population and to reduce the distance for osseous bridging to achieve bony fusion. Preserving as many motion segments as possible by limiting the number of fused segments minimizes alteration of spinal biomechanics and the risk of early degeneration of adjacent segments, and therefore, constitutes a fundamental principle of spinal surgery. Following this principle, several studies [22–26] reported favorable outcome after monosegmental posterior fixation of selected thoracolumbar fractures. However, studies on monosegmental anterior column reconstruction are scarce. Spiegl et al. [27] assessed the clinical and radiological outcome of 14 patients with incomplete burst fractures after a mean of 74 months (range 66–84 months) after thoracoscopic monosegmental ACR using a tricortical iliac crest bone autograft and an additional ventral plate. Nine patients were treated by an anterior only approach and five patients were treated by a combined posterior–anterior approach. Complete (≥ 80%), partial (> 30%), insufficient (≤ 30%) and no fusion at all (0%) was observed in 9, 4, 0 and 1 patient(s), respectively. Mean loss of monosegmental correction accounted for 5.7 ± 2.7°, and ten patients (71%) still reported persistent moderate or intense donor site pain at the iliac crest. Besides donor site morbidity, loss of graft volume and consequent loss of correction is an issue when using bone autografts for ACR. Morrison et al. [28] analyzed the CT scans of 15 patients treated by combined posterior–anterior stabilization and either monosegmental (9 patients) or bisegmental (six patients) ACR using a tricortical iliac crest bone autograft. After a mean of 12.5 months post operation, they observed a mean loss of initial graft volume and length of about 40 and 24%, respectively. The mean postoperative loss of reduction was 12° and was significantly correlated with both loss of graft volume and loss of graft length. The use of a VBRD for ACR in combined posterior–anterior stabilization allows to overcome the shortcomings of bone autografts for ACR, such as loss of bone graft volume, graft fractures and donor-site morbidity. Similarly, postoperative loss of reduction has been reported to be smaller when using a VBRD instead of a bone autograft for ACR in posterior–anterior stabilization [3, 29]. In addition, using a VBRD may be technically less demanding because the height of the expandable VBRD can be easily adjusted to the partial corporectomy defect in situ to restore height and correct sagittal deformity. Knop et al. [2] reported a series of 29 combined posterior–anterior stabilizations of thoracolumbar fractures (T7–L3) using a VBRD. In six patients, the VBRD was implanted monosegmentally. The mean monosegmental surgical correction and monosegmental loss of correction was 18.7° and 1.5°, respectively. Bisegmental kyphosis angles and clinical outcome, however, were not specifically given for monosegmental ACR.

The clinical and radiological outcome observed in the present study is within the range and consistent with that reported in previous studies on combined posterior–anterior stabilization using a VBRD [1–3, 8, 29]. Furthermore, radiological outcome did not significantly differ between monosegmental and bisegmental ACR except for bisegmental loss of correction which was 2.6° greater after monosegmental ACR. On the one hand, however, this difference did not reach significance after exclusion of cases with VBRD subsidence. On the other hand, the clinical relevance of this difference is questionable, as minor differences in surgical correction or loss of correction are typically not reflected by worse clinical outcome [30, 31]. Moreover, about half of the loss of bisegmental correction did not occur at the fused but rather at the intact caudal motion segment (Table 3) and might be the result of an initial overdistraction of this spared segment via posterior reduction and fixation. Similarly, clinical outcome did not significantly differ between the two groups. Patients with monosegmental ACR, however, tended to report a poorer VAS Spine Score and Oswestry Disability Index at final follow-up than those with bisegmental ACR, whereas Roland and Morris Disability Questionnaire and WHOQOL-BREF scores were similar. Although we do not have a definitive explanation for this finding at the moment, the discrepancy between different clinical outcome scores possibly reflects the well-known lack of an appropriately responsive, spinal trauma-specific outcome measurement instrument [32]. Nevertheless, the observed mean loss, for example, in VAS Spine Score after monosegmental ACR was similar in size to that reported by others for bisegmental (or predominantly bisegmental) ACR (16.8 points [3] and 19.7 points [2]).

VBRD subsidence may be a concern in monosegmental ACR since the VBRD is anchored in the caudal part of the fractured vertebra. In fact, five cases of VBRD subsidence were observed in this series after monosegmental but none after bisegmental ACR (*P* = 0.02). In retrospect, the choice of monosegmental ACR was poor in three cases probably because the involvement of the inferior endplate was underestimated. A meticulous analysis of preoperative CT images revealed that the inferior endplate involvement did not only consist of a simple split fracture but rather of multiple fracture lines. In this scenario, the VBRD is positioned onto an inferior endplate whose integrity is severely compromised and axial loading of the VBRD is likely to result in subsidence into the adjacent intervertebral disc (Figs. 2, 3). In the two other cases of VBRD subsidence, the VBRD was placed too far cranially to the inferior endplate of the fractured vertebra and anchored into the weak cancellous vertebral bone (Fig. 4). Consequently, axial loading in an upright position lead to subsidence into the cancellous bone until the inferior endplate was reached. These five cases clearly illustrate the pitfalls of monosegmental ACR: positioning the VBRD (1) onto the weak cancellous bone (i.e., not close enough to the inferior endplate) and (2) onto a sort of “free floating” inferior endplate fragment created by the presence of multiple fracture lines. Both pitfalls can be easily avoided by proper VBRD placement as well as meticulous analysis of preoperative CT images for even subtle fracture lines at the inferior endplate. Interestingly, the VBRD subsidence did not necessarily result in substantially greater loss of correction or worse clinical outcome compared to the mean values of monosegmental ACR without VBRD subsidence (Table 4). Of note, VBRD subsidence was not observed when the VBRD was anchored onto an inferior endplate featuring a single simple split fracture line without significant splaying. In this situation, the VBRD can bridge the single fracture line and can be sufficiently anchored in the adjacent intact parts of the inferior endplate; one single split fracture line, therefore, did not seem to increase the risk for VBRD subsidence and placement thus appears to be safe and reliable.

Posterior instrumentation removal after ACR is another issue that may be debated. In general, the need and potential benefits of implant removal after thoracolumbar fracture stabilization remains controversial and substantially varies between different countries [33–37]. Posterior instrumentation removal after monosegmental ACR, however, is essential to restore mobility in the nonfused segment and to provide the patients with the potentially beneficial effects of sparing one motion segment. In contrast, implant removal after bisegmental ACR does not restore segmental mobility, its benefits are questionable and available evidence is insufficient. Usually it is, therefore, considered only optional, but may be indicated in case of foreign body sensation and persisting discomfort and pain at the posterior spine attributed to local irritation of the muscles by prominent pedicle screw heads. Although we thus routinely recommended implant removal to our patients only after monosegmental but not after bisegmental ACR, half of the patients in the monosegmental group refused removal while about one-third of patients in the bisegmental group strongly wished and demanded implant removal. Anyhow, the potential additional risks of implant removal procedures have to be taken into account already in the initial decision making.

The strengths of this study include the high follow-up rate of 94% (17 of 18 patients who underwent monosegmental ACR), the relatively long mean clinical follow-up period of more than five years and the strict inclusion criteria such as traumatic burst fractures at the thoracolumbar junction (T11–L2) and absence of a neurological deficit. Furthermore, it is the first study to systematically evaluate the feasibility and outcome of monosegmental ACR using a VBRD in combined posterior–anterior stabilization of thoracolumbar burst fractures. Finally, the outcome of monosegmental ACR was related to that of bisegmental ACR using the same thoracoscopic technique, VBRD and aftercare protocol.

Limitations of our study include the relatively small sample size and the retrospective study design. Moreover, fusion was not assessed due to the lack of follow-up CT imaging, and posterior instrumentation was only removed in half of the patients after monosegmental ACR to regain mobility of the spared segment [33]. Finally, when relating the clinical and radiological outcome of monosegmental ACR to that of bisegmental ACR, we were aware that the average degree of vertebral body injury had been greater in the bisegmental than in the monosegmental ACR group. To the best of our knowledge, there are no studies specifically investigating whether the subtype/subgroup of vertebral body burst fracture (subtype A3 vs. A4 according to the AOSpine Thoracolumbar Spine Injury Classification System and subgroup A3.1 vs. A3.2 vs. A3.3 according to the Magerl classification system) affects radiological and clinical outcome after combined posterior–anterior fusion of thoracolumbar burst fractures. Reinhold et al. [3], however, reported that neither the Magerl type of injury (type A vs. B vs. C) nor the group (A1/A2 vs. A3) did significantly affect the bisegmental kyphosis angle (BKA) at final follow-up of 536 operatively treated thoracolumbar fractures (238 combined posterior–anterior, 272 posterior only and 26 anterior only stabilizations). In contrast, preoperative BKA, patient age and level of fracture (thoracic vs. thoracolumbar junction vs. lumbar) were found to significantly affect BKA at final follow-up. As these parameters did not significantly differ between the monosegmental and bisegmental ACR group in our study, relating the outcome of one group to the other was deemed reasonable and valuable to evaluate the short- and mid-term outcome of monosegmental ACR. Nevertheless, future prospective randomized studies with follow-up periods of at least 10 years are needed to clarify whether sparing one spinal segment by monosegmental ACR provides a potential long-term clinical benefit over bisegmental ACR.

## Conclusion

In conclusion, this study indicates that monosegmental ACR using a VBRD is feasible in combined posterior–anterior stabilization of thoracolumbar burst fractures provided that (1) the inferior endplate of the fractured vertebra is intact or (2) features only a single simple split fracture without significant splaying. In our cohort of 37 patients, mean surgical correction and clinical outcome were comparable between monosegmental and bisegmental ACR, whereas bisegmental loss of correction was 2.6° greater in the former than in the latter. Monosegmental ACR allows to preserve one spinal motion segment in this generally young patient population and to reduce the distance for bony fusion. It, therefore, appears to be a desirable option for anterior spinal reconstruction in selected thoracolumbar burst fractures with a largely intact inferior endplate of the fractured vertebra. Surgical pitfalls of monosegmental ACR, however, include VBRD positioning (1) onto the weak cancellous bone (too far cranially to the inferior endplate of the fractured vertebra) and (2) onto a significantly compromised inferior endplate with multiple (even subtle) fracture lines. Ignoring these pitfalls resulted in VBRD subsidence in 5 cases within this series. Proper VBRD positioning close to the inferior endplate as well as meticulous preoperative analysis of inferior endplate involvement on axial CT reconstructions, therefore, are indispensable. In case of suspected additional fracture lines and, of course, in case of obvious inferior endplate comminution, bisegmental ACR should be performed to prevent VBRD subsidence and potential loss of correction.
